# Temporal Trends, Predictors, and Outcomes of Disseminated Intravascular Coagulation in Hospitalizations With Sepsis

**DOI:** 10.7759/cureus.27477

**Published:** 2022-07-30

**Authors:** Dhanshree Solanki, Darshan Lal, Angel Sunny, Xianghui Han, Swathi Iyanar, Abhik Halder, Sanjana Mullangi, Maheshkumar Desai, Uzair Khan, Abhinay Theli, Hiteshkumar Devani, Piyush Kumar, Achint A Patel, Manidhar Lekkala

**Affiliations:** 1 Hospital Administration, Rutgers University, New Brunswick, USA; 2 Hospital Medicine, University of Nevada, Las Vegas, School of Medicine, Las Vegas, USA; 3 Medicine, Jagadguru Sri Shivarathreeshwara (JSS) Medical College, Mysore, IND; 4 Medicine, Hainan Medical University, Haikou, CHN; 5 Medicine, SRM Medical College Hospital and Research Centre, Chennai, IND; 6 Medicine, IQ City Medical College and Narayana Multispecialty Hospital, Durgapur, IND; 7 Internal Medicine, Hillcrest Medical Center, Tulsa, USA; 8 Internal Medicine, Hamilton Medical Center, Medical College of Georgia/Augusta University, Dalton, USA; 9 Internal Medicine, Hospital Corporation of America Healthcare/University of South Florida Morsani College of Medicine Graduate Medical Education/Oak Hill Hospital, Brooksville, USA; 10 Medicine, Guthrie Cortland Hospital, Cortland, USA; 11 Dental Medicine, University of Pittsburgh School of Dental Medicine, Pittsburgh, USA; 12 Medicine, Armed Forces Medical College, Pune, IND; 13 Internal Medicine, Icahn School of Medicine at Mount Sinai, New York City, USA; 14 Oncology, University of Kansas, Kansas City, USA

**Keywords:** dic hospitalizations, outcomes, predictors, dic, sepsis

## Abstract

Background

This retrospective study was conducted to analyze the temporal trends, predictors, and impact of disseminated intravascular coagulation (DIC) on outcomes among septicemic patients using a nationally representative database.

Methods

We derived data from the National Inpatient Sample (NIS) for the years 2008-2017 for adult hospitalizations due to sepsis. The primary outcomes were in-hospital mortality and discharge to facility. The Cochran-Armitage test and multivariable survey logistic regression models were used to analyze the data.

Results

Out of 12,820,000 hospitalizations due to sepsis, 153,181 (1.18%) were complicated by DIC. The incidence of DIC decreased from 2008 to 2017. In multivariable regression analysis, demographics and comorbidities were associated with higher odds of DIC. During the study period, in-hospital mortality among patients with sepsis decreased, but the attributable risk percent of in-hospital mortality due to DIC increased. We observed similar trends for discharge to facility; however, the adjusted odds of discharge to facility due to DIC remained stable over the study period.

Conclusion

Although the incidence of sepsis complicated by DIC decreased, the attributable in-hospital mortality rate due to DIC increased during the study period. We identified several predictors associated with the development of DIC in sepsis, some of which are potentially modifiable.

## Introduction

Sepsis accounts for at least 1.7 million adult cases annually in the USA [1]. Approximately one in three hospitalizations that end in death in a US hospital is associated with sepsis [2]. Sepsis is complicated by disseminated intravascular coagulation (DIC) in about 29%-61% of cases [3-5]. DIC is associated with mortality ranging from 45% to 78% during hospitalizations [6].

DIC is defined by the International Society on Thrombosis and Haemostasis (ISTH) subcommittee as “an acquired syndrome characterized by the intravascular activation of coagulation with loss of localization arising from different causes. It can originate from and cause damage to the microvasculature, which, if sufficiently severe, can produce organ dysfunction” [7,8]. In sepsis-associated DIC, the main event is a systemic inflammatory response to the infectious agent. The causative infectious agent expresses unique cellular pathogen-associated molecular patterns (PAMPs) [9]. PAMPs along with host cell-derived factors are recognized by immune and other host cells. The subsequent activation of intracellular signal transduction pathways leads to the increased production of pro-inflammatory cytokines. This results in the systemic activation of coagulation and the suppression of fibrinolysis, which lead to increased fibrinogen/fibrin degradation products and a prolonged prothrombin time (PT) [9,10]. It also causes simultaneous platelet depletion. These changes lead to a microcirculatory disorder and, subsequently, result in increased severity of sepsis [10].

Currently, there are criteria in place to predict the likelihood of the development of DIC in septic and non-septic patients. The widely accepted scoring systems are the ISTH DIC score and the Japanese Association for Acute Medicine (JAAM) criteria [11,12]. Despite the use of predictors from this scoring system, sepsis patients with DIC continue to have a higher mortality rate when compared to those without DIC [13]. Saito et al. found a significant difference in the rate of diagnosis of DIC in sepsis patients by different scoring systems (61% by JAAM versus 29% by ISTH) [4]. With these findings, we can infer that the laboratory parameters alone are not sufficient to diagnose DIC in sepsis patients.

There is a dearth of contemporary information on the epidemiology and outcomes of DIC in sepsis patients in the USA. We hypothesize that the predictors of DIC in this patient population have not been adequately studied on a large scale. In this retrospective study, we aim to analyze the trends and predictors of DIC in sepsis patients from a large national database to understand its current epidemiology and identify modifiable and non-modifiable predictors.

## Materials and methods

Data sources

We derived our study cohort from the National (Nationwide) Inpatient Sample (NIS) of Healthcare Cost and Utilization Project (HCUP), Agency for Healthcare Research and Quality (AHRQ) [14]. We selected and used datasets from 2008 to 2017. The NIS is one of the largest all-payer publicly available databases on inpatient discharges from US hospitals maintained by the AHRQ [14]. The NIS approximates a 20% stratified sample of discharges from US community hospitals, excluding rehabilitation and long-term acute care hospitals, and contains more than seven million hospitalizations annually [14]. With the established weights in the NIS, this data could be weighted to represent the standardized US population and obtain national estimates with high accuracy [15,16]. As HCUP datasets are de-identified and publicly available, Institutional Review Board (IRB) approval was not needed for our study.

Study population and design

We queried the database between 2008 and 2017 using the International Classification of Diseases, Ninth and 10th Revision, with Clinical Modification diagnosis (ICD-9/10-CM) codes for primary diagnosis of sepsis. We restricted the analysis to only adult and nonpregnant hospitalizations during the study period. We defined patients with DIC using the previously validated diagnosis codes.

Definition of variables

We extracted the baseline characteristics of the study population. Patient-level characteristics included age, sex, race, quartile classification of median household income extrapolated from zip code, and primary payer (Medicare/Medicaid, private insurance, self-pay, or no charge). Hospital-level characteristics included hospital location (urban/rural), hospital bed size (small, medium, and large), region (northeast, midwest or north central, south, and west), and teaching status. We identified comorbid conditions using the Elixhauser Comorbidity Software supplied by HCUP [17].

Statistical analysis

We compared the baseline characteristics of hospitalizations due to sepsis with and without DIC. We used the chi-squared test for categorical variables, Student’s t-test for normally distributed continuous variables, and the Wilcoxon rank-sum test for non-normally distributed continuous variables. To analyze the temporal trends of DIC among sepsis patients, we utilized the Cochran-Armitage trend test and survey linear regression modeling. Additionally, we used survey logistic regression models to estimate the predictors of DIC. Survey logistic regression modeling is an appropriate analysis for data with nested observations, such as the NIS, which is stratified in clusters to produce national estimates [16]. These methods have been used previously for analyses in studies using the NIS dataset [18,19]. The final predictor model was selected after testing for potential interactions and ensuring that there was no multicollinearity between independent variables. We calculated the proportion of yearly in-hospital mortality and discharge to facility. We then calculated attributable in-hospital mortality percent due to DIC and attributable discharge to facility percent due to DIC among sepsis patients. Attributable mortality implies that the deaths would not have occurred had the exposure been absent [20]. Additionally, we also calculated the adjusted odds ratio (aOR) for in-hospital mortality and discharge to facility using a survey logistic regression model after adjusting with confounders. We performed all analyses using designated weight values to produce nationally representative estimates [15]. A two-tailed p-value ≤ 0.05 was considered statistically significant. We used SAS 9.4 (SAS Institute Inc., Cary, North Carolina, USA) for all analyses.

## Results

Baseline characteristics

We analyzed a total of 12,980,000 hospitalizations due to septicemia, out of which 153,181 (1.2%) developed DIC. Most studies done on DIC in sepsis are single-center experiences. The sample size of our study is significantly higher as compared to single-center studies (millions versus thousands) as noted above. Hence, the percentage of DIC in sepsis is comparatively lower in our study. Of the different age groups analyzed, patients in the age range of 50-64 years had a relatively higher percentage of sepsis with DIC (31.1%; p < 0.0001) when compared to patients who had sepsis without DIC (24.2%; p < 0.0001). Moreover, the lowest quartile median household income (30.6% versus 29.5%; p < 0.0001) and the south region (40.2% versus 37.7 %; p < 0.0001) were significantly associated with sepsis with DIC. Among the comorbidities analyzed, fluid and electrolyte disorders (79.5% versus 57.5%) and obesity (27.1% versus 15.4%) showed a higher prevalence in sepsis patients who had DIC than those without DIC. All other comorbidities were more common in sepsis without DIC. Other demographic findings are depicted in Table 1.

**Table 1:** Baseline characteristics of the study population † This represents a quartile classification of the estimated median household income of residents in the patient’s zip code. These values are derived from the zip code demographic data obtained from Claritas. The quartiles are identified by values of 1-4, indicating the poorest to wealthiest populations. Because these estimates are updated annually, the value ranges vary by year. AIDS: acquired immunodeficiency syndrome; PUD: peptic ulcer disease; HMO: health maintenance organization

Characteristics	Septicemia without DIC	Septicemia with DIC	Total	p-value
Overall	12,820,000	153,181	12,973,181	
Age in years (mean ± SE)	66.4 ± 0.04	62.7 ± 0.1		<0.0001
Age in years (median (q1-q3))	68 (55-80)	63 (51-75)		<0.0001
Age in years (%)				<0.0001
18-34	6.2	7.2	6.2	
35-49	10.4	13.1	10.5	
50-64	24.2	31.1	24.2	
65-79	32	31.1	32	
≥80	27.3	17.6	27.2	
Gender (%)				<0.0001
Male	48.5	47.2	48.5	
Female	51.4	52.8	51.5	
Race (%)				<0.0001
White	66.5	57.5	66.4	
Black	12.6	16	12.6	
Hispanic	8.9	11.1	9	
Others	5.8	8.5	5.9	
Missing	6.1	6.9	6.2	
Comorbidities (%)				
Obesity	14	10.3	14	<0.0001
Hypertension	59.4	43.3	59.2	<0.0001
Diabetes mellitus with chronic complications	13.3	8.6	13.3	<0.0001
Diabetes mellitus without chronic complications	21.6	15.4	21.5	<0.0001
Congestive heart failure	22.6	23.8	22.7	<0.0001
Valvular heart disease	6.9	6.7	6.9	<0.0001
History of chronic pulmonary disease	27.5	17.6	27.4	<0.0001
Pulmonary circulatory disease	4.2	6.7	4.2	<0.0001
Peripheral vascular disease	8.8	10.9	8.8	<0.0001
Paralysis	7.1	5.5	7.1	<0.0001
Solid tumor without metastasis	4	4.2	4	<0.0001
Lymphoma	1.9	3.4	1.9	<0.0001
Metastatic cancer	4.8	7.9	4.8	<0.0001
Weight loss	15.4	27.1	15.5	<0.0001
Liver disease	6.1	17.2	6.2	<0.0001
Alcoholism	4.2	8.1	4.2	<0.0001
Other neurological disorders	15.8	11	15.8	<0.0001
Renal failure	23.6	24.7	23.6	0.17
Hypothyroidism	13.9	9.5	13.9	<0.0001
Arthritis	4	3.6	4	<0.0001
Anemia chronic blood loss	1	1.9	1	<0.0001
Anemia deficiency	30.2	27.7	30.1	<0.0001
Fluid and electrolyte disorders	57.5	79.5	57.8	<0.0001
Depression	11.8	6.4	11.8	<0.0001
Psychoses	5.3	3.8	5.3	<0.0001
Drug abuse	3.9	4.6	3.9	<0.0001
AIDS	0	0.1	0	<0.0001
PUD	0.5	0.8	0.5	<0.0001
Median household income† (%)				<0.0001
First quartile	29.5	30.6	29.6	
Second quartile	25.7	24.4	25.7	
Third quartile	23.4	22.5	23.3	
Fourth quartile	23.4	22.5	19.3	
Primary insurance (%)				<0.0001
Medicare/Medicaid	76.6	70	76.5	
Private including HMO	17.2	21.4	17.2	
Uninsured/self-pay	6.1	8.4	6.1	
Hospital bed size (%)				<0.0001
Small	16.4	11.8	16.4	
Medium	28.5	26.2	28.4	
Large	54.8	61.5	54.9	
Hospital type (%)				<0.0001
Rural	11.3	6.1	11.2	
Urban non-teaching	34.9	34.1	34.9	
Teaching	53.5	59.3	53.6	
Hospital region (%)				<0.0001
Northeast	17.8	15.5	17.7	
Midwest	21.3	17.4	21.3	
South	37.7	40.2	37.7	
West	23.3	26.9	23.4	
Day of admission				0.0089
Weekday	73.7	73.4	73.7	
Weekend	26.3	26.6	26.3	
Source of admission (%)				<0.0001
Transfer from other hospital or other health facility	16.1	22.1	16.2	
Emergency department	83.9	77.9	83.8	
Type of admission (%)				<0.0001
Emergent or urgent	96.2	96	96.2	
Elective	3.8	4	3.8	

Temporal trends of DIC among sepsis patients 

**Figure 1:**
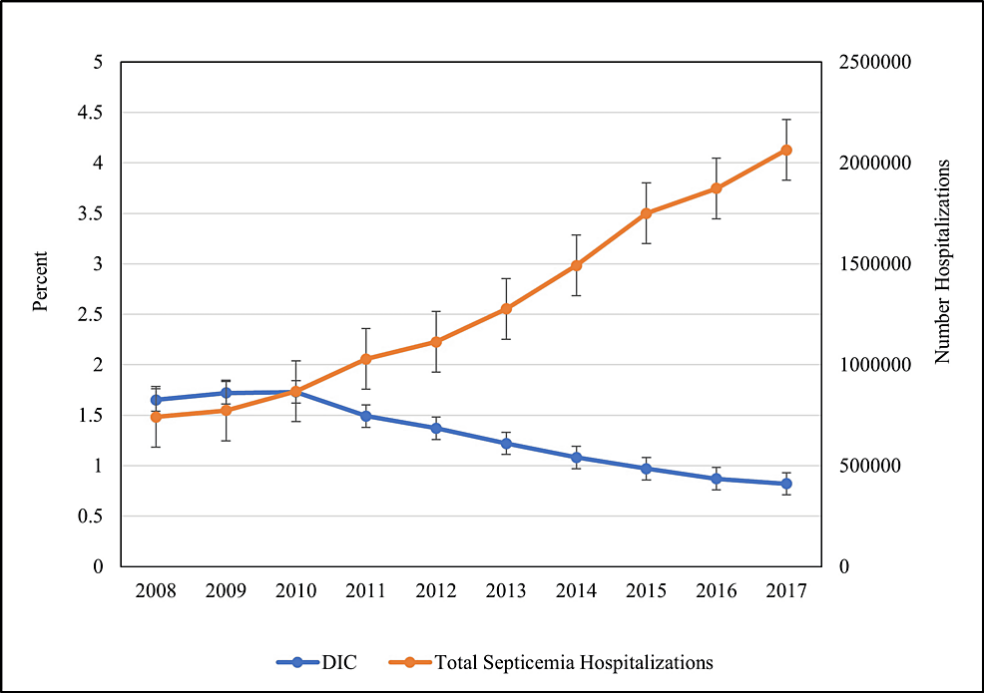
Temporal trends of DIC among sepsis patients DIC: disseminated intravascular coagulation

The prevalence of DIC decreased from 1.6% in 2008 to 0.8% in 2017 with 6% decrease (OR: 0.94; 95%CI: 0.93-0.94; p < 0.001) over the study years (Figure 1).

Predictors of DIC among sepsis patients

We found that several predictors were associated with an increased likelihood of developing DIC in septicemia patients. These predictors include female sex (OR: 1.21; 95%CI: 1.17-1.24; p < 0.0001) and uninsured/self-pay patients (OR: 1.19; 95%CI: 1.13-1.25; p < 0.0001) along with comorbid conditions such as pulmonary circulatory diseases (OR: 1.23; 95%CI: 1.17-1.29; p < 0.0001), congestive heart failure (OR: 1.09; 95%CI: 1.06-1.13; p < 0.0001), liver disease (OR: 2.37; 95%CI: 2.27-2.46; p < 0.001), and weight loss (OR: 1.25; 95%CI: 1.21-1.29; p < 0.0001). Meanwhile, there were also a few predictors that were found to have reduced risk in developing DIC, such as age ≥65 (OR: 0.85; 95%CI: 0.81-0.90; p < 0.0001), White race (OR: 0.78; 95%CI: 0.75-0.81; p < 0.0001), obesity (OR: 0.75; 95%CI: 0.72-0.79; p < 0.0001), diabetes mellitus (OR: 0.74; 95%CI: 0.70-0.77; p < 0.0001), and hypertension (OR: 0.66; 95%CI: 0.64-0.68; p < 0.0001). Other predictors associated with DIC in septicemia patients are listed in Table 2.

**Table 2:** Predictors of DIC among sepsis patients † This represents a quartile classification of the estimated median household income of residents in the patient’s zip code. These values are derived from the zip code demographic data obtained from Claritas. The quartiles are identified by values of 1-4, indicating the poorest to wealthiest populations. Because these estimates are updated annually, the value ranges vary by year. HMO: health maintenance organization; CI: confidence interval; LL: lower limit; UL: upper limit

Independent variable/characteristic	Odds ratio (95%CI)	p-value
Year	0.94 (0.93-0.94)	<0.0001
Age		
18-34	0.97 (0.92-1.03)	0.31
35-49	Referent	
50-64	0.95 (0.90-1.00)	0.07
≥65	0.85 (0.81-0.90)	<0.0001
Gender (%)		
Male	Referent	
Female	1.21 (1.17-1.24)	<0.0001
Race (%)		
White	0.78 (0.75-0.81)	<0.0001
Black	Referent	
Hispanic	0.94 (0.89-1.00)	0.05
Others	1.09 (1.02-1.16)	0.008
Comorbidities (%)		
Obesity	0.75 (0.72-0.79)	<0.0001
Hypertension	0.66 (0.64-0.68)	<0.0001
Diabetes mellitus	0.74 (0.70-0.77)	<0.0001
Congestive heart failure	1.09 (1.06-1.13)	<0.0001
Valvular heart disease	1.05 (0.99-1.10)	0.09
History of chronic pulmonary disease	0.61 (0.59-0.63)	<0.0001
Pulmonary circulatory disease	1.23 (1.17-1.29)	<0.0001
Peripheral vascular disease	1.47 (1.41-1.53)	<0.0001
Paralysis	0.69 (0.65-0.73)	<0.0001
Metastatic cancer	1.64 (1.56-1.71)	<0.0001
Weight loss	1.25 (1.21-1.29)	<0.0001
Liver disease	2.37 (2.27-2.46)	<0.0001
Alcoholism	0.98 (0.93-1.04)	0.56
Anemia deficiency	0.79 (0.77-0.82)	<0.0001
Drug abuse	0.93 (0.88-0.99)	0.02
Other neurological disorders	0.68 (0.65-0.71)	<0.0001
Renal failure	1.28 (1.24-1.33)	<0.0001
Arthritis	1.05 (0.98-1.12)	0.13
Electrolyte and fluid disorders	2.14 (2.07-2.22)	<0.0001
Lymphoma	1.66 (1.55-1.78)	<0.0001
Median household income† (%)		
First quartile	0.92 (0.87-0.97)	0.002
Second quartile	0.92 (0.87-0.96)	0.0005
Third quartile	0.93 (0.89-0.97)	0.001
Fourth quartile	Referent	
Primary insurance (%)		
Medicare/Medicaid	Referent	
Private including HMO	1.18 (1.14-1.22)	<0.0001
Uninsured/self-pay	1.19 (1.13-1.25)	<0.0001
Hospital bed size (%)		
Small	0.83 (0.78-0.88)	<0.0001
Medium	0.90 (0.85-0.95)	0.0001
Large	Referent	
Hospital type (%)		
Rural	0.71 (0.65-0.77)	<0.0001
Urban non-teaching	0.91 (0.86-0.96)	0.0006
Teaching	Referent	
Hospital region (%)		
Northeast	0.75 (0.70-0.81)	<0.0001
Midwest	0.75 (0.70-0.81)	<0.0001
South	0.96 (0.90-1.02)	0.18
West	Referent	
Day of admission		
Weekday	Referent	
Weekend	1.01 (0.99-1.04)	0.37
Source of admission (%)		
Transfer from other hospital or other health facility	1.26 (1.21-1.32)	<0.0001
Emergency department	Referent	
Type of admission (%)		
Emergent or urgent	Referent	
Elective	0.95 (0.88-1.02)	0.15

In-hospital outcomes of DIC among sepsis patients

**Figure 2:**
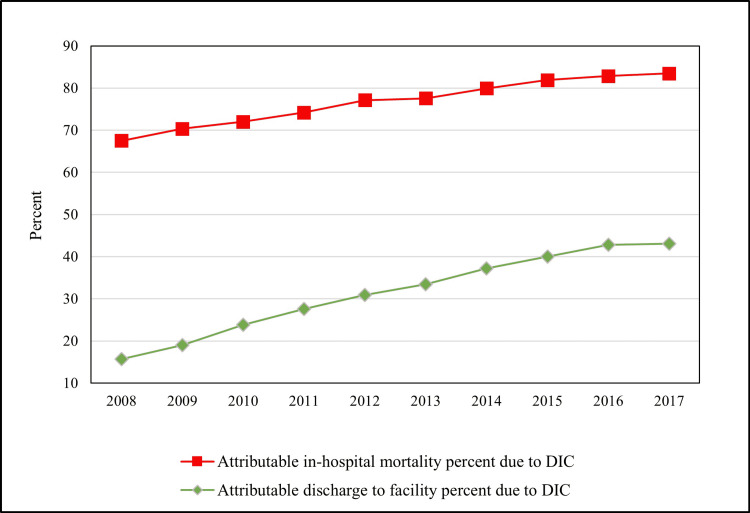
Trends of outcomes of sepsis hospitalizations complicated by DIC DIC: disseminated intravascular coagulation

The overall unadjusted in-hospital mortality was significantly higher in sepsis patients with DIC (54.1 versus 11.7%; p < 0.0001). Also, these patients had less frequent discharges to facility (27.5% versus 34.7%; p < 0.0001) and home (18.4% versus 53.6%; p < 0.001) when compared to sepsis patients without DIC. After adjusting for demographics, hospital-level characteristics, Deyo modification of the Charlson Comorbidity Index, and concurrent diagnoses, the adjusted in-hospital mortality remained significantly higher in sepsis patients with DIC than in those without (aOR: 3.93; 95%CI: 3.78-4.08; p < 0.001). In addition, sepsis patients with DIC had higher odds of discharge to facility (aOR: 1.54; 95%CI: 1.46-1.61; p < 0.0001) than at home. During the period from 2008 to 2017, there was a steady increase in attributable in-hospital mortality percent due to DIC, which increased from 67.5% in 2008 to 83.5% in 2017. Attributable discharge to facilities due to DIC went up from 15.7% in 2008 to 43.1% in 2017 (Figure 2). In the trend analysis, the adjusted odds of in-hospital mortality increased from 2.55 (95%CI: 2.23-2.91; p < 0.001) in 2008 to 4.81 (95%CI: 4.33-5.35; p < 0.0001) in 2017. The adjusted overall odds of discharge to facility remained stable from 1.35 (95%CI: 15-1.58; p < 0.001) in 2008 to 1.63 (95%CI: 1.40-1.89; p < 0.001) in 2017.

## Discussion

This study analyzed the temporal trends in developing DIC in septicemia patients from 2008 to 2017 using an HCUP database of 12,973,181 adult hospitalized patients. We noted that DIC complicated 1.18% of all sepsis admissions. The hospitalizations due to sepsis increased from 2008 to 2017; however, the incidence of sepsis complicated by DIC decreased. This trend could be due to improvement in healthcare delivery secondary to approaches such as early goal-directed therapy and Surviving Sepsis Campaign (SSC) and early recognition of sepsis. Singh et al. similarly noted that the incidence of DIC decreased from 2004 to 2010 in ICU patients [6].

In a study on adult patients with septic shock admitted to a medical ICU, Kelm et al. noted a median age of 63 years for patients who developed DIC during their hospital stay [8]. Similarly, in our study, we noted that the median age for sepsis hospitalizations with DIC was 63 years. The elderly population is susceptible to infection (due to decreased T-cell and B-cell activity) and chronic medical conditions [21]. Additionally, loss of collagen, increased skin tears at a later age of life, reduced cytokine release with age, and decreased neutrophil phagocytic activity make them more susceptible to infections and sepsis [22]. We found that White Americans are at a lesser risk of developing DIC compared to African Americans (OR: 0.78; 95%CI: 0.75-0.81; p < 0.0001). African Americans have higher circulating levels of VWF, factor VIII, and fibrinogen. Higher circulating VWF levels may be due to increased baseline production by endothelium or reduced clearance [23]. This could be a likely explanation.

As noted, before, there are standardized scoring systems to predict the likelihood of the development of DIC in sepsis patients. In this study, one of our goals was to find other parameters that could predict the likelihood of developing DIC in septicemia patients. Our results show that certain non-modifiable factors such as male sex, Caucasian race, and age ≥ 65 years were found to have a reduced risk of developing DIC. We hypothesize that many patients ≥ 65 years die before they develop DIC, and thus, our analysis shows a reduced risk of developing DIC in the elderly despite the elderly being at higher risk for infections. While many comorbidities were associated with an increased likelihood of the development of DIC in septicemia patients, such as congestive heart failure, pulmonary circulatory disease, weight loss, and liver disease, comorbidities such as hypertension, diabetes mellitus, and obesity were unexpectedly found to have a reduced likelihood of the development of DIC in septicemia patients. It was surprising to see that patients with diabetes mellitus had a reduced likelihood of developing DIC in septicemia patients. In Japan, there was a 2017 study that partially supports our finding of the reduced incidence of DIC in septicemia patients with diabetes mellitus [24]. In this study population of septicemic patients, it was noted that diabetic patients were at lesser odds of developing DIC than nondiabetic patients. Stratified analysis was performed on the different medications that diabetic patients were taking. It was found that the recent use of certain oral hypoglycemic drugs was significantly associated with a lower risk of DIC. However, overall, the study also stated that diabetes mellitus was associated with a higher risk of DIC, particularly when it had been treated recently with insulin [24]. It can be inferred that perhaps the oral hypoglycemic drugs could have a protective effect on sepsis patients. Kothari et al. noted that oral hypoglycemic agents exert anti-inflammatory effects by modulating inflammatory pathways, independent of their antihyperglycemic activity [25].

Patients with hypertension were also found to have a reduced rate of DIC in sepsis patients. While endotoxins and tumor necrosis factor-α (TNF-α) activate the coagulation system through the extrinsic route, the intrinsic route of coagulation (contact-system dependent) also gets activated and seems to be involved in the induction of hypotension and the activation of the fibrinolytic system [26]. Pixley et al. studied the relationship between hypotension and the development of DIC in the baboon model. The baboons were infused with *Escherichia coli* to produce lethal bacteremia with hypotension. This study suggested that irreversible hypotension correlates with prolonged activation of the contact system, which could theoretically contribute to the DIC associated with septicemia [27]. With this information, we hypothesize that existing hypertensive disorder could possibly prevent sepsis patients from developing DIC. However, this hypothesis needs to be tested in future studies. In our study, it was found that obesity had reduced the likelihood of the development of DIC in septicemic patients. At the same time, patients with weight loss were found to have an increased likelihood of developing DIC. This finding is surprising considering that obesity is usually linked with a higher risk of other comorbidities. A 2013 study reported that crude hospital mortality of obese and very obese patients with septic shock was lower than normal weight patients [28]. While this study is specific about mortality rather than a prediction of DIC, it can be inferred that obesity might have a preventative effect on the development of DIC in septicemia patients. More robust evidence is needed to confirm or refute this finding. As expected, many comorbidities are associated with an increased likelihood of DIC and mortality in sepsis patients, such as liver disease, malignancy, and renal failure. A multicenter Japanese study noted that the main underlying diseases associated with DIC were infections, solid tumors, and hematologic malignancies [29]. The underlying diseases with the highest mortality rates in DIC patients were infections, trauma/burn, cardiovascular diseases, and digestive diseases [29]. Additionally, we noted that the odds of developing DIC in sepsis were higher in patients transferred from other hospitals or other health facilities than those admitted from the emergency department (OR: 1.26; 95%CI: 1.21-1.32; p < 0.0001). This is likely because the patients transferred to a hospital with a higher level of care are sicker and have a higher incidence of comorbidities.

We noted that the adjusted in-hospital mortality was significantly higher in sepsis patients with DIC than in those without (aOR: 3.93). In addition, sepsis patients with DIC were associated with more frequent discharges to facility (aOR: 1.54). The higher mortality rate among septicemia patients with DIC in our study is similar to those of some previous studies. In a large randomized trial of 1,690 patients with severe sepsis, placebo‐treated patients with DIC had a much higher 28‐day mortality of 43% compared with 27.1% in placebo‐treated patients without DIC [30]. In another large randomized clinical trial of 2,314 patients with severe sepsis, placebo-treated patients with DIC were at higher risk of death than patients without DIC. This was true for mortality rates at 28 days (40% versus 22.2%; p = 0.004) and at 90 days (50.4% versus 32.9%; p = 0.002) [31]. A multicenter prospective survey of severe sepsis in Japan conducted by the JAAM Sepsis Registry Study Group also reported higher in-hospital mortality of 38% compared with 22% in septic patients without DIC [12].

During the study period from 2008 to 2017, we noted that the in-hospital mortality rate in sepsis with DIC increased from 52.3% to 55.2%. Importantly, the attributable in-hospital mortality percent due to DIC increased from 67.5% in 2008 to 83.5% in 2017. From 2008 to 2017, discharge to facility in sepsis patients with DIC increased from 56.1% to 61.4%. In addition, attributable discharge to facility percent due to DIC increased from 15.7% in 2008 to 43.1% in 2017. The trends of in-hospital mortality in DIC patients have been variable across different studies. A single-center, population-based, retrospective cohort study conducted in Mayo Clinic ICUs in the USA including 154 patients with DIC from 2004 to 2010 has shown that the mortality rate remains the same over the decades. There was a nonsignificant trend for a decrease in in-hospital mortality over time, from 58% in 2004 to 45% in 2010. However, this study was conducted in a single center [6]. Thus, its result cannot be generalized. A nationwide retrospective observational study of 325,327 adult DIC patients using a Japanese database showed that the overall 28-day mortality for DIC patients decreased from 41.8% to 36.1% over the eight-year study period from July 2010 to March 2018 [32]. Compared with prior studies, the upward trend in in-hospital mortality of DIC over years in our study could be due to differences in case selection (ISTH criteria or ICD-10-CM code versus ICD‐9‐CM plus ICD-10-CM code), time period under consideration, and more selective (single center/region) versus nationally representative population. Although we were not able to identify specific causes for the upward trend in in-hospital mortality of DIC, the large study sample that we used and national-level statistics guarantee the credibility of the experimental results.

Our study has several notable strengths. First, this is the first multiyear US population-based study from a nationally representative sample. Therefore, these results are generalizable. Second, our study does not suffer from the bias seen with single-center studies, which frequently either lack scientific rigor or external validity [33]. Third, the NIS database includes patients from ICU and non-ICU settings. Hence, there is no concern for falsely low DIC incidence rate, which could likely be seen in studies that are confined to ICU settings.

There are some important limitations to our study. We excluded children, which precludes generalizing the study results to pediatric patients with concurrent sepsis and DIC. The NIS database is an administrative database prone to coding errors. Despite these limitations, we believe that the large sample size of our study and in-depth statistical analysis have equipped us with a clearer understanding of this important health issue.

## Conclusions

Female and uninsured patients were noted to be at higher odds of developing DIC during sepsis as were patients with pulmonary circulatory diseases, congestive heart failure, renal failure, and liver disease. The prevalence of hospitalizations with sepsis increased from 2008 to 2017; however, the prevalence of sepsis complicated by DIC decreased. Mortality in patients admitted with sepsis decreased from 2008 to 2017. However, the attributable in-hospital mortality rate due to DIC increased in sepsis hospitalizations during the same time period. Future studies are needed to understand the potential factors causing increased mortality in sepsis patients with DIC.
